# Effects of dietary faba bean (*Vicia faba* L.) inclusion on carcass characteristics, meat quality, and blood biochemistry in Awassi lambs

**DOI:** 10.14202/vetworld.2025.1863-1869

**Published:** 2025-07-11

**Authors:** Belal S. Obeidat, Nawras L. Al Shabuol, Linda Alyahya

**Affiliations:** 1 Department of Animal Production, Faculty of Agriculture, Jordan University of Science and Technology, P.O. Box 3030, Irbid, 22110, Jordan; 2 Discipline of Clinical Pharmacy, School of Pharmaceutical Sciences, Universiti Sains Malaysia, 11800, Pulau Pinang, Malaysia

**Keywords:** Awassi lambs, blood parameters, carcass traits, faba bean, meat quality, protein alternative

## Abstract

**Background and Aim:**

The search for sustainable and cost-effective protein alternatives to soybean meal in livestock diets has led to the exploration of legumes such as faba beans [FBs] (*Vicia faba* L.). This study investigated the effects of dietary inclusion of FBs on carcass traits, meat quality, and selected blood parameters in Awassi lambs.

**Materials and Methods:**

Twenty-four male Awassi lambs (initial body weight: 20.54 ± 0.798 kg) were randomly assigned to two groups (n = 12 per group) and fed isonitrogenous diets either with no FB (control diet [CON]) or 20% FB on a dry matter basis (FB20) for 70 days. The trial consisted of a 7-day acclimation period, followed by 63 days of data collection. Carcass evaluation, meat quality analysis (pH, water-holding capacity [WHC], color, and shear force), and blood biochemistry profiling were performed.

**Results:**

FB20 supplementation significantly increased loin weight and lean content (p < 0.05), while reducing subcutaneous and total fat percentages (p < 0.05). WHC improved (p = 0.024), shear force decreased (p = 0.024), and meat redness (a*) significantly increased (p < 0.0001) in FB20 lambs. No differences were observed in other meat quality parameters such as pH, cooking loss, whiteness (L*), and yellowness (b*) values. The crude protein content in meat was higher in the FB20 group (p = 0.043), indicating an improved nutritional value. Blood biochemical markers, including urea nitrogen, glucose, cholesterol, creatinine, liver enzymes, and lipoproteins, did not differ between groups (p > 0.05), indicating no adverse health effects.

**Conclusion:**

The inclusion of 20% FB in the diet of Awassi lambs improved carcass composition and meat quality without negatively affecting animal health. The observed improvements in meat tenderness, protein content, and color, along with unchanged blood profiles, support the use of FBs as a viable and sustainable protein source in lamb production. Future studies should investigate the long-term effects, consumer acceptance, and optimal inclusion rates across various breeds and production systems.

## INTRODUCTION

One of the primary challenges faced by livestock breeders is the high cost and limited availability of feed. In particular, soybean meal – the predominant legume used in sheep diets – is both expensive and limited in availability, creating a need for alternative feed sources [1]. As a result, legumes such as peas [2], chickpeas [3], and faba beans (FB) [4] have been explored as viable substitutes.

FBs (*Vicia faba* L.) are widely cultivated and economically accessible in the Mediterranean region. As an annual legume [5], FB is rich in nutrients, notably crude protein (CP) (30% on a dry matter [DM] basis) and essential amino acids, making it a promising alternative to soybean meal [6, 7]. Recent research by Al Shabuol and Obeidat [4] has demonstrated that incorporating 20% FB into the diet of Awassi lambs can enhance growth performance and reduce the cost of weight gain by partially replacing soybean meal and barley grain.

Numerous studies have examined the effects of FB inclusion on carcass characteristics, i.e., one study by Surra *et al*. [8] reported no significant impact on carcass weight in Rasa Aragonesa lambs fed FB. Conversely, another study by Lestingi *et al*. [9] found that lambs fed FB had higher carcass weights than those fed lupine seeds. In addition, the study observed a higher loin percentage in lambs receiving FB compared to those fed lupine or a combination of legumes. Facciolongo *et al*. [10] similarly reported greater carcass weights in FB-fed lambs relative to those fed soybean meal, although no differences were noted in warm dressing percentage, cold dressing percentage, or the distribution of carcass components (e.g., neck, steaks, brisket, shoulder, loin, abdomen, leg, and testicle) between the FB and soyb-ean meal groups.

Lanza *et al*. [11] investigated the substitution of soybean meal with 38% FB and found no differences in carcass weight, muscle conformation, fatness, or fat color. In Barbarine lambs, Boukhris *et al*. [12] reported that those receiving 20% FB had a higher net dressing percentage compared to lambs fed 10% FB or soybean meal. Moreover, Purroy *et al*. [13] observed that higher dietary FB protein levels (12%, 15%, and 18% DM) were associated with reduced omental and mesenteric fat. They also noted lower overall carcass fat deposition in lambs fed FB compared to those fed soybeans or lentils.

Despite the growing interest in utilizing alternative protein sources in small ruminant nutrition, research on the direct impact of FB (*V. faba* L.) inclusion on carcass traits, meat quality, and physiological responses in Awassi lambs remains limited. Previous studies [8–13] have explored the substitution of soybean meal with various legumes such as peas, lupines, and chickpeas, yielding mixed results in terms of carcass composition and growth performance. While some research has indicated potential benefits of FB in reducing fat deposition or improving dressing percentage, many of these studies were conducted on breeds other than Awassi or did not simultaneously assess a compr-ehensive range of carcass, meat, and biochemical para-meters. Furthermore, the data on how FB affects meat quality traits such as water-holding capacity (WHC), shear force, and meat color in lambs are still fragmented and inconclusive. Importantly, limited attention has been paid to the physiological safety of FB inclusion, with an evaluation of its impact on blood biochemical markers and liver enzyme profiles in growing lambs. This knowledge gap hampers the ability of farmers and nutritionists to make informed decisions about integrating FB into feeding strategies for Awassi lambs under real-world production conditions.

This study was designed to evaluate the effects of incorporating 20% FB (on a DM basis) into the diet of growing Awassi lambs, with a focus on three core domains: Carcass characteristics, meat quality, and blood biochemical parameters. Specifically, the rese-arch aimed to determine whether partial replacement of conventional protein sources (i.e., soybean meal and barley) with FB could enhance economically and nutritionally relevant traits, such as loin weight, lean-to-fat ratio, and meat tenderness, without compromising animal health or performance. By integrating detailed carcass measurements, meat physicochemical analyses, and a broad panel of blood biomarkers, this study aimed to gain a comprehensive understanding of the benefits and limitations of FB inclusion in lamb nutrition. The findings are expected to provide evidence-based guid-ance for sustainable and cost-effective feeding practices in sheep production systems, particularly in regions where high feed input costs and limited soybean availa-bility are prevalent.

## MATERIALS AND METHODS

### Ethical approval

All procedures were approved by the Institut-ional Animal Care and Use Committee of Jordan University of Science and Technology (JUST) (Protocol #: 16/03/02/495).

### Study period and location

The study was conducted from November 2020 to January 2021 at the Animal Farm of the Agricultural Research and Training Unit, Faculty of Agriculture, JUST.

### Experimental design

Descriptions of the methods and information on performance and intake are based on the study of Al Shabuol and Obeidat [4]. In a completely randomized design, 24 lambs (body weight [BW] = 20.54 ± 0.798 kg) were divided into two groups and fed two isonitrogenous meals (16.4% CP and DM basis). Animals were stratified by initial body weight and randomly assigned to die-tary treatment according to a completely randomized design. FB was administered at 0 (control diet [CON diet]) and 20% (FB20) concentrations of dietary DM to partially replace barley and soybean meal. During the study, diets were mixed biweekly in a farm feed mill and sampled after mixing to ensure consistency in their chemical composition. The health status of the lambs was checked, and they were weighed, ear-tagged, and treated for internal parasites (2 mL/lamb; Ivermectin 1%, Ivermic, Laboratorios Microsules Uruguay S.A., Uruguay). During the feeding period, the health and welfare of the lambs were monitored daily.

### Feeding management

The 70-day trial was divided into 63 days for data collection (i.e., carcass features, meat quality, and blood parameters) and 7 days for acclimation (i.e., for the pen and diets). Animals were acclimatized using experimental diets by controlling the amount of food offered. The lambs were housed individually in shaded, concrete pens (0.75 × 1.5 m), each equipped with a plastic waterer (7 L) and a feeder (10 L). The pens were designed to provide natural ventilation and were cleaned every 2 weeks or as needed to maintain their dryness and cleanliness throughout the study. During the experiment, the experimental diets were provided twice daily at 8:00 a.m. and 3:00 p.m. Diets were given *ad libitum* (110% of the previous day’s intake) to the lambs as a total mixed ration diet (Table 1). Refusals were collected daily, recorded before the morning feeding, and subsampled for each lamb; they were then frozen for later analysis. At the end of the study, the refusal was composited for each lamb, and its nutrient content was analyzed. Feed intake was calculated by subtracting the amount of feed refused from the amount provided. Fresh water was available *ad libitum* throughout the experimental period. Lambs on the FB20 diet consumed more DM, CP, neutral detergent fiber, acid detergent fiber, and ether extract (EE) than those on the CON diet. The BWs of the two diets were similar [4]. Lambs fed the FB20 diet had a higher average daily gain (229.9 g) than the CON diet (195.8 g). Lambs fed the FB20 diet had lower feed costs (US$/1000 kg, 319 vs. 365) and gain costs (US$/kg of gain, 1.63 vs. 1.98) than those fed the CON diet [4].

**Table 1:** Ingredients and chemical composition of diets-containing FBs fed to Awassi lambs.

Item	Diets^a^	

	CON	FB20
Ingredients (% DM)		
Barley grain	53.0	39.0
Soybean meal	18.0	12.0
FBs^b^	0	20.0
Wheat straw	27.0	27.0
Salt	1.0	1.0
Limestone	0.9	0.9
Vitamin-mineral premix^c^	0.1	0.1
Nutrients (% DM)		
DM	90.5	90.8
CP	16.3	16.4
Neutral detergent fiber	29.1	31.3
Acid detergent fiber	19.0	19.4
EE	1.9	2.4

a The diets were the CON and 20% FB (FB20) of dietary DM.

b Nutrient content in the faba beans were 92.5, 23.4, 27.2, 10.4, and 4.3% for dry matter, crude protein, neutral detergent fiber, acid detergent fiber, and ether extract, respectively.

c Composition per kg contained (vitamin A, 600,000 IU; vitamin D3, 200,000 IU; vitamin E, 75 mg, vitamin K3, 200 mg; vitamin B1, 100 mg; vitamin B5, 500 mg; lysine 0.5%; DL-methionine, 0.15%; manganese oxide, 4000 mg; ferrous sulfate, 15,000 mg; zinc oxide, 7000; magnesium oxide, 4000 mg; potassium iodide, 80 mg; sodium selenite, 150 mg; copper sulfate, 100 mg; cobalt phosphate, 50 mg, and dicalcium phosphate, 10,000 mg. FB=Faba bean, CON = Control diet, DM=Dry matter, CP=Crude protein, EE=Ether extract

### Blood analysis

On days 1, 30, and 60 of the experiment, blood samples were drawn from each lamb’s jugular vein using a simple vacutainer before feeding at 8:00 a.m. Blood samples were centrifuged at 1,008 × *g* for 30 min at 4°C to separate the serum. After that (i.e., 30 min), the serum samples were kept in tubes at –20°C until they were analyzed. The concentrations of blood bioch-emical parameters, including triglycerides, creatinine, low-density lipoprotein (LDL), high-density lipoprotein (HDL), cholesterol, urea nitrogen content, and serum glucose, were measured. In addition, liver enzymes, including aspartate aminotransferase (AST), alkaline phosphatase (ALP), and alanine aminotransferase (ALT), were measured. All analyses were performed using commercial kits (Biolabo S.A.S., Less Hautes Rivers, Maizy, France) and a spectrophotometer (JENWAY 6105 UV/Vis, Model 6105, Jenway LTD, Felsted, Dunmow, Essex, CM6 3LB, UK). The average intra- and inter-assay coefficients of variation were 3.5% and 3.4%, respectively. The reading was performed in duplicate, and the machine was calibrated before the reading. All blood serum samples were analyzed in duplicate.

### Sample collection

Lambs were taken to slaughterhouses after the trial period. The animals were slaughtered through exsanguination following an 18-h fast in accordance with established halal procedures [14]. To determine chilling losses and dressing percentage, the liver, kidneys, lungs, trachea, spleen, and other non-carcass parts were removed and weighed. Both immediately following slaughter (hot carcass weight) and after refrigeration for 24 h at 4°C (cold carcass weight), the carcass weight was recorded. One day after slaughter, measurements were taken of the longissimus dorsi muscles and chilled carcasses to evaluate the linear dimensions of leg fat depth (L3), fat depth (C), eye muscle depth (B), eye muscle width (A), tissue depth (GR), rib fat depth (J), and shoulder fat depth (S2). After cutting and dissecting, the right leg and loin were promptly vacuum-packed and stored at −20°C until further measurements. The longissimus muscle was removed and frozen at −20°C for further analysis.

### Meat quality analysis

The frozen longissimus muscle was thawed over-night at 4°C, and samples were collected to assess the meat quality parameters of the muscle. WHC [15], cooking loss [16], shear force [17], pH, and meat color (as measured by a colorimeter) were measured in triplicate. Color was assessed using the CIELAB color system. Color coordinates (Lab*) were quantified using slices of each 15-mm-thick thawed muscle. Color slices were wrapped in an oxygen-permeable film and stored at 4°C for 2 h before measurement, allowing for surface oxygenation. The color coordinates were measured in triplicate using a handheld colorimeter (12 MM Aperture U 59730-30, Cole-Parameter International, Accuracy Microsensors Inc., Pittsford, NY, USA).

The cooking loss was calculated using duplicate slices (25 mm thick) after the raw slices were weighed and cooked for 90 min (until they reached an internal temperature of 72°C) in plastic bags in a water bath at 75°C. The weight of each slice was measured after pathing to determine the proportion of water removed. The cooked slices were stored in the chiller at 4°C for 24 h to measure the shear force or softness. A Warner-Bratzler shear blade with a triangular slot cutting edge, mounted on a Salter Model 235, was used to cut six 1 cm³ cores from the slices and shear them perpendicular to the direction of the muscle fibers. Weighing the peak force (kg) required to shear the cores was one method for determining the tenderness of meat. The shear force measurement was done in triplicate. The pH of the muscle was determined using a pH spear (pH spear, large screen, waterproof pH/temperature tester, double injection, model 35634-40, Eurotech Instruments, Malaysia) on a homogenate consisting of 10 mL of neutralized 5-mM iodoacetate reagent and 2 g of raw meat. Before measuring the pH, the pH probe calibration was performed. The water-retaining capacity was assessed using the techniques outlined by Grau and Hamm [15]. According to the Association of Official Analytical Chemists [18] procedures, a slice of meat sample was analyzed in duplicate for DM (determined at 100°C in an air-forced oven for 24 h; method 967.03), CP (determined using the Kjeldahl procedure; method 955.04), and EE (crude fat; method 922.06).

### Statistical analysis

The Statistical Analysis System (SAS) mixed procedure was used to analyze data variance. The random variable was animal. The animal nested in diet was the residual error in the statistical model, which included diet as a fixed variable and lamb as a random effect. Data normality and homogeneity of variance were assessed using the UNIVARIATE procedure in SAS (Version 9.4, SAS Institute Inc., Cary, NC, USA). The adjusted p-value was used to compare means using Tukey’s test to determine pairwise differences. p ≤ 0.05 was considered statistically significant.

## RESULTS

### Carcass characteristics

As shown in Table 2, FB20 lambs exhibited a significantly greater loin weight (575 g) compared to CON lambs (470 g; p = 0.019), along with a higher percentage of lean tissue (p = 0.037). In addition, lambs fed FB showed significantly lower subcutaneous fat percentage and total fat percentage (p ≤ 0.034) than those in the CON group. However, no other carcass characteristics were altered by FB inclusion in the diet (p ≥ 0.171).

**Table 2:** Effects of feeding FB on carcass, non-carcass components, carcass cut weights, percentages, and dissected loins of Awassi lambs.

Item	Diet^a^			

	CON (n = 12)	FB20 (n = 12)	SEM	p-value
Fasting live weight (kg)	32.58	34.04	1.277	0.437
Hot carcass weight (kg)	15.70	16.38	0.658	0.483
Cold carcass weight (kg)	14.54	15.33	0.701	0.441
Dressing percentage	44.47	45.00	0.701	0.629
Non-carcass components (kg)	1.208	1.271	0.0376	0.265
Carcass cut weight (kg)	12.36	13.16	0.505	0.286
Fat tail (kg)	1.43	1.33	0.154	0.653
Loin weight (g)	470	575	27.0	0.019
Intermuscular fat (g/100 g)	2.75	2.39	0.206	0.192
subcutaneous fat (g/100 g)	12.76	9.67	0.841	0.025
Total fat (g/100 g)	15.51	12.13	0.9906	0.034
Total lean (g/100 g)	50.69	53.73	0.931	0.037
Total bone (g/100 g)	22.84	24.24	1.166	0.401
Meat-to-bone ratio	2.32	2.25	0.158	0.763
Meat: fat ratio	3.70	4.44	0.353	0.171

a The diets were the CON and 20% FB (FB20) of dietary DM. FB=Faba bean, CON = Control diet, DM=Dry matter, SEM=Standard error of the mean

### Carcass linear dimensions

According to Table 3, no significant differences (p ≥ 0.4) were observed in the carcass linear measu-rements, including L3, GR, J, A, B, C, or S2, between the two groups.

**Table 3:** Effects of feeding FBs on carcass leaner dimensions of Awassi lambs.

Item	Diet^a^			

	CON (n = 12)	FB20 (n = 12)	SEM	p-value
L3 (mm)	1.75	2.04	0.275	0.416
GR (mm)	9.63	9.70	0.530	0.922
J (mm)	1.75	1.75	0.230	1.000
A (mm)	51.08	49.71	1.0100	0.395
B (mm)	22.96	22.33	0.846	0.612
C (mm)	1.17	1.29	0.1215	0.482
S2 (mm)	1.4583	1.2917	0.1842	0.535

a The diets were the CON and 20% FB (FB20) of dietary DM. FB=Faba bean, CON = Control diet, DM=Dry matter, SEM=Standard error of the mean, L3=Leg fat depth, GR=Tissue depth, J=Rib fat depth, A=Eye muscle width, B=Eye muscle depth, C=Fat depth, S2=Shoulder fat depth

### Meat quality parameters

As shown in Table 4, FB20 lambs exhibited improved meat quality traits, including higher WHC (p < 0.05), lower shear force (p < 0.05), and significantly greater redness (a*) values (p < 0.0001) compared to the CON group. However, no differences were detected in pH, cooking loss, or color coordinates whiteness (L*) and yellowness (b*) (p ≥ 0.169).

**Table 4:** Effects of feeding FBs on meat quality of Awassi lambs.

Item	Diet^a^			

	CON (n = 12)	FB20 (n = 12)	SEM	p-value
pH	5.87	5.85	0.008	0.169
Cooking loss (g/100 g)	38.6	38.2	0.3562	0.322
WHC (g/100 g)	22.9	23.0	0.9403	0.024
Shear force (kg/cm^2^)	7.11	5.57	0.4912	0.024
Color coordinates				
L*	30.7	30.8	0.80	0.908
a*	6.96	13.88	0.5308	< 0.0001
b*	20.87	20.32	0.407	0.349

a The diets were the CON and 20% FB (FB20) of dietary DM. FB=Faba bean, CON = Control diet, DM=Dry matter, SEM=Standard error of the mean, pH=Potential of hydrogen, WHC=Water-holding capacity, L*=Whiteness, a*=Redness, b*=Yellowness

### Meat chemical composition

As indicated in Table 5, meat DM, EE, and ash content were not significantly affected by the FB20 diet (p ≥ 0.283). However, CP content was significantly higher in the meat of lambs fed FB20.

**Table 5:** Effects of feeding FBs on the chemical composition of meat in Awassi lambs.

Item	Diet^a^			

	CON (n = 12)	FB20 (n = 12)	SEM	p-value
DM	26.29	25.79	0.315	0.283
CP	21.68	22.05	0.1771	0.043
EE	2.41	2.18	0.207	0.445
Ash	1.51	1.54	0.041	0.586

a The diets were the CON and 20% FB (FB20) of dietary DM. FB=Faba bean, CON = Control diet, DM=Dry matter, SEM=Standard error of the mean

### Blood biochemical parameters

Table 6 presents the blood profile results, sho-wing that dietary FB inclusion had no significant effects on serum levels of urea nitrogen, glucose, creatinine, cholesterol, HDL, LDL, AST, ALT, or ALP in growing Awassi lambs.

**Table 6:** Effects of feeding FBs on the blood parameters of Awassi lambs.

Item	Diet^a^			

	CON (n = 12)	FB20 (n = 12)	SEM^b^	p-value
Blood urea nitrogen (mg/dL)	24.0	21.9	1.24	0.122
Glucose (mg/dL)	53.4	58.6	3.48	0.165
Cholesterol (mg/dL)	57.4	58.6	3.99	0.584
HDL (mg/dL)^c^	29.6	26.8	2.28	0.239
LDL (mg/dL)^c^	24.7	25.3	3.16	0.865
Triglycerides (mg/dL)	18.2	18.3	1.85	0.930
Creatinine (mg/dL)	0.95	1.02	0.071	0.365
AST ( IU/L)^c^	23.6	23.8	3.40	0.943
ALT (IU/L)^c^	9.2	8.4	2.32	0.752
ALP (IU/L)^c^	92.0	107.5	21.42	0.484

a The diets were the CON and 20% FB (FB20) of dietary DM. FB=Faba bean, DM=Dry matter,

b SEM=Standard error of the mean; CON = Control diet,

c HDL=High-density lipoprotein, LDL=Low-density lipoprotein, AST=Aspartate aminotransferase, ALT=Alanine aminotransferase, ALP=Alkaline phosphatase

## DISCUSSION

This study aimed to evaluate the effects of incorporating 20% FB into the diet of growing Awassi lambs on carcass characteristics, meat quality traits, and blood biochemical parameters, with the goal of assessing FB as a sustainable alternative protein source to soybean meal.

Consistent with the hypothesis of the study, the current results confirmed that overall carcass characteristics and meat quality were similar between the two diets. However, the inclusion of 20% FB in the diets of lambs improved loin weight and muscle percentage and decreased subcutaneous and total fat percentages. In addition, blood metabolites and liver enzymes did not differ between the CON and FB diets. Overall, our results demonstrated that feeding growing Awassi lambs a diet containing 20% FB did not adv-ersely affect their body composition or health status.

### Carcass composition and comparisons with literature

Carcass characteristics such as intermuscular fat percentage, total bone percentage, meat-to-bone ratio, and meat-to-fat ratio did not change due to FB inclu-sion. These observations align with previous findings by Lestingi *et al*. [9], who reported no differences in slau-ghter body weight, empty body weight, chilling weight, and dressing percentage among FB, lupin, and peas. Similarly, Polidori *et al*. [19] reported no change in hot carcass weight and dressing percentage in FB-fed lambs.

Interestingly, our study revealed increased loin weight and total lean percentage, which contrasts with the findings of Lestingi *et al*. [9], who reported no differences in loin weight between protein sources. Moreover, they found that lambs fed FB had lower lean-to-fat ratios and higher fat content in loin cuts compared to those fed lupin or pea – findings opposite to ours, where FB decreased subcutaneous and total fat percentages. This improvement in loin weight suggests enhanced muscle growth, likely promoted by the high protein and amino acid content, particularly lysine, of FB [19, 20]. These amino acids are key drivers of muscle growth and development. In addition, the reduction in fat accumulation indicates that FB contributes to producing carcasses with better muscle conformation and leaner profiles, aligning with the current consumer demand for low-fat meat. The fatty acid profile in FB [21] may also play a role in reducing adiposity while enhancing muscle development.

### Carcass linear dimensions and lean distribution

Our findings showed no significant differences in carcass leaner dimensions between the FB and CON groups, which is in agreement with Lestingi *et al*. [9]. Their leg dissection analysis showed similar patterns across diets with soybean meal, FB, or lupin. The abse-nce of differences in L3, J, eye muscle dimensions, and other carcass size indicators confirms that FB enhances lean mass without causing disproportionate fat depo-sition. This outcome is valuable for producers aiming to improve meat yield and quality without compromising production efficiency.

### Meat quality traits: pH, water retention, and tenderness

Our results indicated no change in ultimate meat pH due to FB inclusion, consistent with previous studies on both lambs and poultry-fed legume-based diets [19, 22, 23]. Stable postmortem pH values indicate that FB does not negatively impact muscle energy meta-bolism at slaughter, thereby preserving meat integrity.

The WHC increased in the FB20 group, a favorable outcome, as higher WHC correlates with juicier meat. This differs from previous broiler studies, where WHC remained unaffected by FB [22, 23]. Improved WHC in lambs may be attributed to the dietary fiber and protein matrix in FB that influence muscle microstructure and moisture retention [24]. In contrast, cooking loss remained unchanged, consistent with the findings of Polidori *et al*. [19].

Shear force was significantly lower in the FB20 group, suggesting improved tenderness. Although Polidori *et al*. [19] found no differences in shear strength between FB and soybean meal-fed lambs, our results suggest that FB may influence postmortem proteolysis and fiber structure [25], contributing to greater meat softness, a key quality attribute valued by consumers.

### Meat color attributes

In our study, a* increased in meat from FB-fed lambs, whereas L* and b* values were unaffected. These results partially align with previous findings by Polidori *et al*. [19] and Milczarek and Osek [23], which reported no changes in meat color across dietary treatments in both broilers and lambs. However, one study in poultry noted an increase in leg meat lightness with FB diets [22].

Increased a* may be linked to higher myoglobin concentration and improved oxidative muscle status induced by the antioxidants in FB. Meat color is a critical visual quality parameter that affects consumer perception, and greater a* values suggest an enhanced market appeal of FB-fed lamb meat.

### Meat chemical composition

No significant changes were observed in meat DM, EE, or ash content with FB20 inclusion, consistent with earlier studies on both poultry and lamb [19, 22]. However, CP content was significantly higher in the FB20 group, in contrast with Polidori *et al*. [19] and Kuźnicka *et al*. [22], who reported no differences in protein levels. This finding supports the hypothesis that FB, due to its high protein density, can improve the nutritional profile of lamb meat. From a consumer standpoint, higher protein content enhances the meat’s dietary value and appeals to health-conscious markets.

### Blood biochemical response and health status

The inclusion of FB did not affect any of the evaluated blood parameters (serum urea nitrogen, glucose, creatinine, cholesterol, HDL, LDL, AST, ALT, or ALP), confirming its metabolic neutrality. This indicates that the FB diet met the nutritional and physiological requirements of growing lambs without inducing sys-temic stress or metabolic imbalance.

Given the high protein content of FB, the unch-anged blood urea nitrogen levels between groups suggest efficient nitrogen metabolism and protein utilization. Lambs were able to assimilate dietary protein without excessive nitrogen excretion, reinforcing the metabolic suitability of FB. These findings suggest that FB can serve as a safe and nutritionally sound protein source, particularly in environments where soybean meal is expensive or scarce.

## CONCLUSION

The inclusion of 20% FB in the diet of growing Awassi lambs improved key carcass traits and meat quality parameters without negatively affecting animal health or performance. Notably, lambs fed FB20 exhibited increased loin weight and lean percentage, reduced subcutaneous and total fat content, improved WHC, enhanced a* in meat color, and decreased shear force, indicating improved tenderness. Additionally, the CP content of meat was significantly higher in the FB-fed group, thereby enhancing its nutritional value. Blood biochemical parameters, including liver enzy-mes and markers of nitrogen metabolism, remained unaffected, confirming the physiological safety of FB supplementation.

From a practical standpoint, these findings support the use of FB as a cost-effective and sustainable alternative protein source to soybean meal in lamb production, particularly in regions where feed costs are a constraint. The economic advantages, including reduced feed and gain costs, further underscore its value in commercial operations.

The study’s strengths lie in its comprehensive evaluation of carcass composition, meat physicochemical properties, and blood biochemistry within a controlled and replicable design. However, limitations include the relatively short feeding duration and the focus on a single breed under specific environmental conditions, which may limit broad generalization.

Future research should explore the long-term effects of FB inclusion on reproductive performance, immune response, and sensory meat attributes. Studies across different breeds, feed systems, and inclusion levels are warranted to optimize recommendations and assess consumer acceptance of FB-fed lamb products.

In conclusion, incorporating 20% FB into Awassi lamb diets offers a viable strategy to improve meat quality and carcass leanness without compromising animal health, thereby supporting the sustainable intensification of small ruminant production.

## AUTHORS’ CONTRIBUTIONS

BSO NLA, and LA: Designed and conducted the study and drafted the manuscript. All authors have read and approved the final manuscript.
